# Postoperative Hemodynamic Instability Following Adrenalectomy for Presumed Adrenocortical Carcinoma: Anesthetic Implications of a Rare Cavernous Hemangioma

**DOI:** 10.7759/cureus.95754

**Published:** 2025-10-30

**Authors:** Abbas Merchant, Haashim Rahman, Aaryan Patel, Ishan Deshmukh, Andy Burk, Zaheer Irani, Constantino G Lambroussis

**Affiliations:** 1 Anesthesiology, Lake Erie College of Osteopathic Medicine, Erie, USA; 2 Physical Medicine and Rehabilitation, Lake Erie College of Osteopathic Medicine, Erie, USA; 3 Vascular Surgery, Lake Erie College of Osteopathic Medicine, Elmira, USA; 4 Anesthesiology, Albany Medical College, Albany, USA; 5 Medicine, Rochester General Hospital, Rochester, USA; 6 Osteopathic Medicine/Family Medicine, Lake Erie College of Osteopathic Medicine, Elmira, USA

**Keywords:** adrenal tumor, early postoperative complication, hemangioma, large intra‑abdominal mass, pocus (point of care ultrasound, rare vascular tumor, retroperitoneal tumor, shock

## Abstract

Adrenal cavernous hemangiomas are rare, often large, benign vascular lesions that can mimic adrenal malignancy on imaging. Their perioperative management poses significant anesthetic challenges, particularly regarding hemodynamic stability and intraoperative bleeding risk. We describe a 77-year-old with a 17 cm right retroperitoneal mass presumed to be adrenocortical carcinoma, presenting with vague abdominal discomfort. Cross-sectional imaging showed a large, heterogeneous, hypervascular mass displacing the right kidney, leading to surgical resection. Intraoperatively, the mass was highly vascular, requiring transfusion and vasopressor support. Postoperatively, the patient developed refractory hypotension, prompting an urgent but ultimately unnecessary re-exploration, which revealed no active bleeding. Final pathology confirmed a benign adrenal cavernous hemangioma with thrombosis and infarction. The patient recovered with supportive care and was discharged on hospital day seven. This case underscores the diagnostic challenges in distinguishing benign from malignant adrenal masses preoperatively and highlights the risk of hemodynamic instability following resection of large vascular tumors. It also illustrates the potential for avoidable reoperation in the absence of definitive evidence of hemorrhage. The case emphasizes the utility of perioperative point-of-care ultrasound (POCUS) for rapid assessment of volume status and cardiac function, which may aid in differentiating causes of postoperative hypotension and guide targeted management, potentially avoiding unnecessary re-exploration.

## Introduction

Renal cavernous hemangiomas are incredibly rare, benign vascular malformations, typically found incidentally during imaging [1]. These tumors are composed of dilated vascular spaces and channels, and due to their vascular nature and often large size, can be misdiagnosed as an adrenocortical carcinoma (ACC) [2,3]. In certain cases, these highly vascularized tumors can rupture and be misdiagnosed as an abdominal aortic aneurysm rupture due to the amount and rate of exsanguination [4].

Since it was first discovered and classified in 1955, only 67 reports of this rare adrenal pathology have been reported [5]. Like other adrenal masses, the adrenal cavernous hemangioma is typically found incidentally on other forms of imaging [5]. While the majority of incidental adrenal lesions are benign, a thorough diagnostic evaluation is crucial to differentiate them from malignant processes, particularly given the potential for misdiagnosis with adrenal carcinomas or metastatic disease [6]. This rarity, coupled with their often asymptomatic nature, frequently leads to their incidental discovery during imaging for unrelated conditions, thereby posing a diagnostic challenge due to their similarity to other adrenal pathologies [7]. The differential diagnosis for adrenal masses is extensive and includes adenomas, myelolipomas, pheochromocytomas, and metastatic lesions, making the definitive identification of cavernous hemangiomas particularly challenging given their nonspecific imaging features and absence of hormonal activity [8,9]. Consequently, a comprehensive understanding of their imaging characteristics, although often atypical, is vital for accurate diagnosis and appropriate management [10]. The intraoperative and anesthetic implications involved in resection of such a complex adrenal mass include vasodilation, hypovolemia, myocardial dysfunction, and adrenal insufficiency. This case report aims to highlight the diagnostic complexities and management considerations of adrenal cavernous hemangioma, emphasizing the role of advanced imaging and biopsy in achieving a definitive diagnosis for this exceedingly rare adrenal tumor.

## Case presentation

A 77-year-old male with a history of well-controlled hypertension and no significant cardiac history was referred to our institution for evaluation of a large right-sided abdominal mass. The patient reported several weeks of vague right upper quadrant discomfort but denied weight loss, hormonal symptoms, flushing, palpitations, or signs of endocrine excess. Cross-sectional imaging had been initiated for the evaluation of abdominal discomfort.

**Figure 1:**
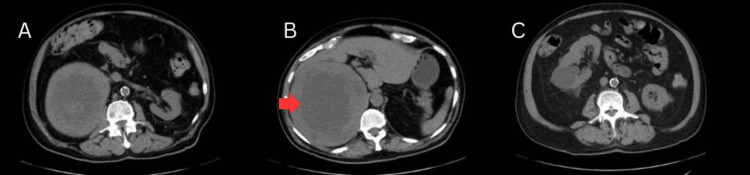
Axial CT image of a 17-cm adrenal hemangioma demonstrating a region of central infarction. Axial CT images of the abdomen demonstrating a 17-cm heterogeneous mass arising from the right retroperitoneum (A-B). The mass displaces the right kidney anteriorly and demonstrates central low attenuation with peripheral enhancement (C), features concerning for malignancy such as adrenocortical carcinoma or necrotic sarcoma. An area of central hypoattenuation is noted by the red arrow, which was later determined to be an area of infarction.

Initial computed tomography (CT) images of the abdomen revealed a well-circumscribed, heterogeneous mass measuring 17.0 cm in maximum diameter located in the right retroperitoneum. The mass displaced the right kidney anteriorly and demonstrated peripheral arterial enhancement with central hypoattenuation, raising suspicion for a necrotic or hemorrhagic neoplasm. The imaging impression included adrenocortical carcinoma as the leading consideration, with differential diagnosis including retroperitoneal sarcoma or hemorrhagic adrenal lesion (Figures 1A-1C).

**Figure 2:**
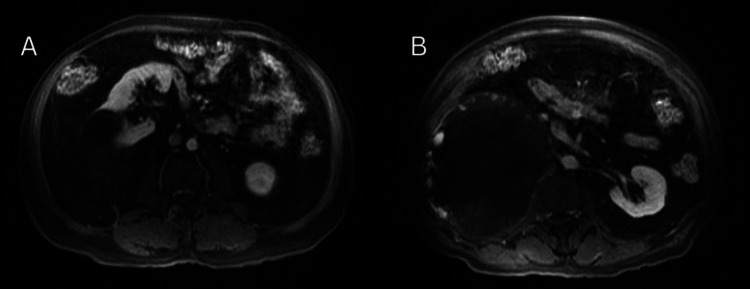
Axial MRI of Adrenal Hemangioma Axial MRI T1-weighted (A) and post-contrast sequences with fat saturation (B) showing a large hypointense retroperitoneal mass with peripheral nodular enhancement. The adrenal origin is favored. The imaging appearance, while nonspecific, remained concerning for a primary adrenal malignancy.

A subsequent MRI of the abdomen was ordered to further characterize the lesion. The mass was hyperintense on T1-weighted sequences with scattered punctate areas of hyperintensity, suggesting hemorrhagic components. It exhibited irregular but gradual peripheral enhancement without evidence of vascular invasion. The right adrenal gland appeared compressed against the anterior aspect of the mass, supporting an adrenal origin. No metastatic disease or lymphadenopathy was identified (Figures 2A-2B).

Endocrinologic evaluation, including plasma metanephrines, aldosterone, renin, and 8 a.m. cortisol, was all within normal limits, effectively ruling out a functional adrenal tumor. Given the lesion's size (>6 cm), imaging features, and the inability to exclude malignancy, surgical resection was recommended. The patient was deemed a reasonable surgical candidate after preoperative optimization. A multidisciplinary surgical and anesthesiology team planned an open adrenalectomy with intraoperative vigilance for potential bleeding or catecholamine surge.

Intraoperatively, standard ASA monitors and an arterial line were placed, and general anesthesia was induced without complications. A thoracic epidural catheter had been inserted preoperatively for anticipated postoperative pain control. Through a midline laparotomy, a large retroperitoneal mass was visualized adherent to the lateral aspect of the adrenal gland. Dissection was complicated by adherence to surrounding retroperitoneal structures and compression of the renal vasculature. Estimated blood loss was 800 mL, and the patient received two units of packed red blood cells along with crystalloid resuscitation. A norepinephrine infusion was initiated intraoperatively to maintain mean arterial pressures between 65-70 mmHg during tumor manipulation.

**Figure 3:**
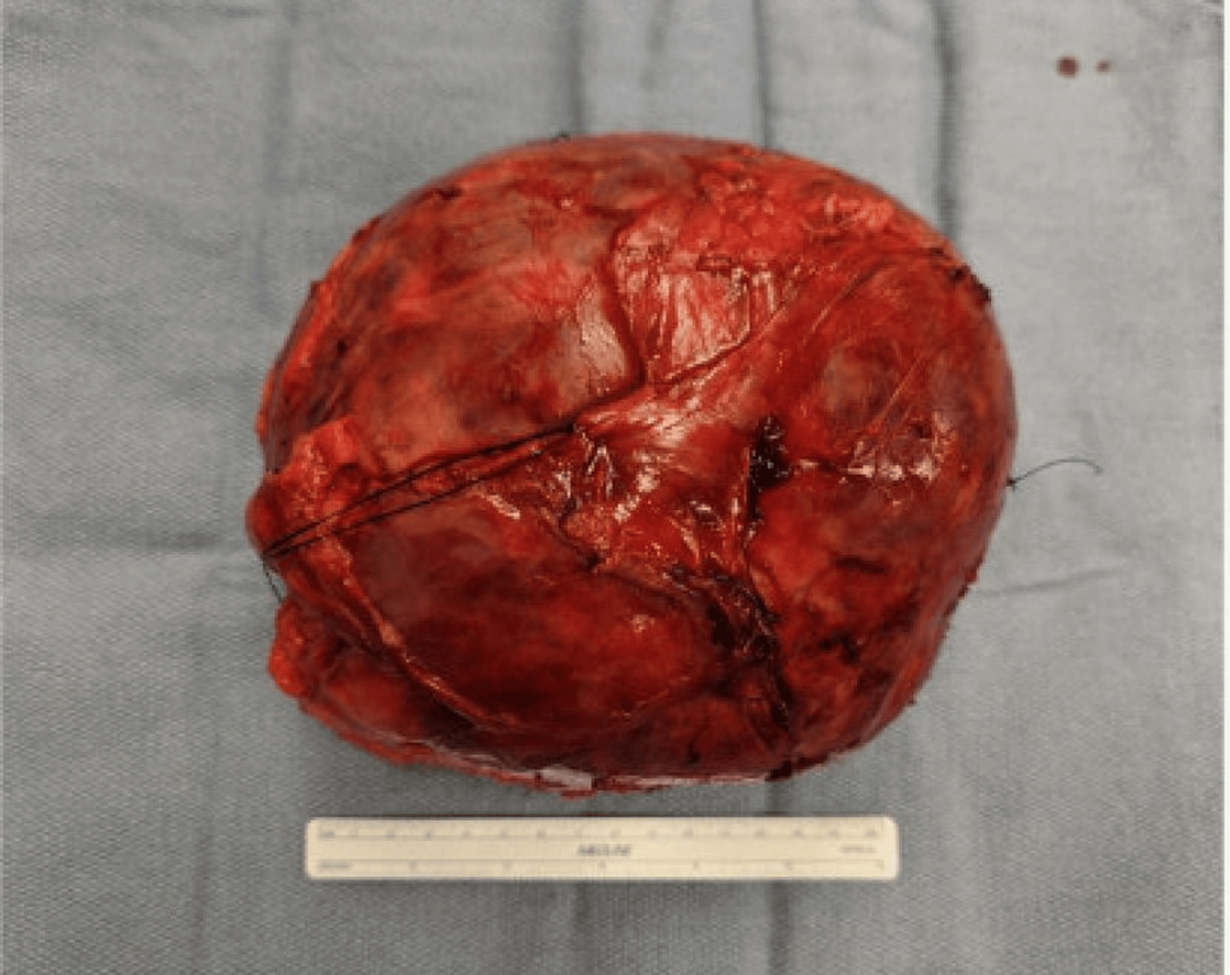
Gross specimen of a 17-cm adrenal cavernous hemangioma with a fibrous capsule. Gross specimen of the resected 17-cm adrenal mass showing a dark red, vascular lesion with a fibrous capsule. Histopathologic examination later confirmed a cavernous hemangioma with central infarction.

The mass was successfully removed en bloc with the right adrenal gland. Incidentally, the appendix, which appeared fibrotic and tethered within the surgical field, was also removed prophylactically. The surgical team described the tumor as unusually vascular, with areas of dark hemorrhagic discoloration. Gross specimen photography revealed a large, encapsulated, dark red mass with external vascular markings (Figure 3). 

Following surgery, the patient was transferred to the post-anesthesia care unit (PACU), intubated, and remained on a low-dose norepinephrine infusion. Within 30 minutes, the patient developed refractory hypotension (blood pressure (BP) 85/50 mmHg), despite fluid resuscitation and vasopressor titration. He remained responsive and appropriately sedated, and an epidural infusion had not yet been started. Laboratory evaluation showed a lactate of 4.0 mmol/L with mild metabolic acidosis, and hemoglobin was stable compared to end-of-case values (Table 1). The surgical site showed no external signs of bleeding. Nevertheless, the decision was made to return to the operating room urgently for concerns of intra-abdominal hemorrhage.

**Table 1:** Postoperative laboratory values.

	Value	Reference range
Lactate	4 mmol/L	<2 mmol/L
Hemoglobin	9 g/dL	13.5-17.5 g/dL

During re-exploration, no active bleeding was identified. There was moderate blood-tinged ascitic fluid in the tumor bed, but all surgical ligatures remained intact. No expanding hematoma or arterial hemorrhage was found. The abdominal cavity was irrigated, and the incision was re-closed. The patient was transferred to the ICU postoperatively for vasopressor-dependent hypotension and a temperature of 35 °C (95 °F). He was actively rewarmed, and over the next 18 hours, hemodynamics improved with ongoing fluid administration and tapering vasopressors. He was extubated on postoperative day 1 and transferred to the surgical ward by day 3.

Final surgical pathology revealed a benign adrenal cavernous hemangioma with areas of thrombosis and infarction, with no evidence of malignancy or atypia. The appendix showed a benign serrated polyp without dysplasia. The patient recovered without further complications and was discharged on hospital day 7, tolerating diet, ambulating, and off vasopressors.

This case exemplifies the perioperative complexity of large adrenal tumors of uncertain origin and highlights the risks of hemodynamic instability when the underlying physiology is not fully understood. It also underscores how intraoperative and PACU-based point-of-care ultrasound (POCUS) might have assisted in determining volume status or cardiac function before pursuing reoperation.

## Discussion

In this case, the failure to recognize the true cause of hypotension led to avoidable re-operation. We discuss the anesthetic implications of large adrenal masses with unclear hemodynamic profiles and review the utility of perioperative POCUS. Recent literature highlights that POCUS allows anesthesiologists to rapidly determine shock states, volume status, and cardiac function, enabling targeted interventions [11,12]. We also outline the added risks incurred by an unnecessary exploratory surgery, including hypothermia, blood loss, infection, fluid overload, and prolonged ICU stay, and how these might have been mitigated with better hemodynamic guidance.

This case highlights several important considerations in the perioperative management of large adrenal tumors with unclear hemodynamic behavior. First, it underscores the rarity of adrenal cavernous hemangioma and the difficulty in differentiating such benign lesions from malignant adrenal tumors preoperatively. Adrenal hemangiomas are typically incidental findings and are very uncommon; most reported cases are large (often >10 cm) and unilateral [13]. In our patient, the 17 cm tumor was presumed to be an ACC given its size and radiologic features. Indeed, surgical resection was appropriate because definitive diagnosis of adrenal masses usually requires histopathology, and resection both prevents potential hemorrhage and confirms whether a malignancy is present [13]. Only after resection was the true benign nature of the lesion revealed. In hindsight, knowing it was a hemangioma explains its highly vascular nature and possible contribution to intraoperative blood loss; however, unlike a pheochromocytoma, it did not cause any preoperative hypertension or tachycardia, and unlike an ACC, it produced no hormones.

The central lesson from this case is the critical need for thorough hemodynamic evaluation when faced with unexpected instability. The patient’s postoperative hypotension was ultimately not due to surgical bleeding, but rather likely due to a combination of factors: relative hypovolemia (from blood loss and third-space fluid shifts), vasodilation (from anesthetics and epidural analgesia), and possibly reduced cardiac output in an elderly patient with diastolic dysfunction. Unfortunately, the immediate assumption was that bleeding was the cause, given the recent major surgery. This led to an emergent re-exploration that was ultimately unnecessary. Such re-operations carry significant risk and cost. The patient was exposed to additional anesthetic time and surgical trauma, which in turn increased the risk of complications like hypothermia. The second operation led to a drop in core temperature, which is known to increase risks of coagulopathy, wound infection, and cardiac events [14]. There was also additional blood loss, as reopening a surgical site can cause oozing and requires disruption of forming clots, potentially increasing total blood loss. Infection risk increased as a return to the operating room breaks the sterile field a second time and prolongs exposure, raising the likelihood of surgical site infection or pneumonia. Fluid overload was a concern as aggressive fluid resuscitation in response to hypotension (before and during re-exploration) can lead to edema, pulmonary congestion, or abdominal compartment issues once it becomes clear there was no hemorrhagic loss. Lastly, the patient had a prolonged ICU stay. Each of the above factors contributed to a more protracted critical care course (our patient stayed in ICU two days largely due to the sequelae of re-operation). Indeed, re-exploration after major surgery is associated with longer hospital stays and higher rates of morbidity [15].

Many of these adverse outcomes might have been avoided with a more nuanced assessment of the patient’s hemodynamic status in the PACU. This is where POCUS could offer substantial value. A bedside ultrasound examination by the anesthesia or critical care team might have identified the true origin of the hypotension without delay. A focused cardiac ultrasound exam (also known as a focused transthoracic echocardiography or *quick echo*) could have been performed within minutes. If this exam had shown, for example, a small, hyperdynamic left ventricle and an empty, collapsing IVC, it would strongly suggest hypovolemia or *tank* failure as the cause of hypotension rather than hemorrhagic shock [16]. Conversely, if a large pericardial effusion or poor ventricular contractility were seen, other specific interventions could be directed. In our patient, an IVC ultrasound view in PACU almost certainly would have shown significant collapsibility (indicating insufficient preload). Additionally, a focused assessment with sonography in trauma (FAST) examination of the abdomen could have been performed to evaluate for free fluid. The absence of intra-abdominal fluid on ultrasound would argue against a large internal bleed as the cause of shock. Together, these POCUS findings might have reassured the team that conservative management (fluids, vasopressors, and blood transfusion if needed) was the appropriate course, obviating the immediate need to reopen the incision.

Beyond the postoperative period, one can argue that preoperative POCUS assessment might have also been beneficial. Given the patient’s advanced age and history of hypertension, it is plausible that he had some degree of diastolic dysfunction (a stiff left ventricle with dependence on higher filling pressures) [16]. A focused echocardiographic examination before induction of anesthesia could have identified features such as left ventricular hypertrophy or abnormal relaxation patterns. Recognition of such a cardiac phenotype would alert the anesthesiologist that the patient might not tolerate aggressive vasodilation or rapid blood loss without volume support. For instance, Bughrara et al. emphasize that POCUS can pinpoint the precise cause of hemodynamic instability and guide targeted therapy [11]. They describe how a hypertrophic, *thick* left ventricle is highly sensitive to hypovolemia and can even develop dynamic left ventricular outflow tract obstruction if underfilled [11]. In such cases, inotropic drugs can be detrimental, whereas maintaining adequate preload is crucial [11]. Applying this knowledge, if our patient had evidence of a thickened myocardium on POCUS, the anesthetic plan could have been adjusted to prioritize volume maintenance (for example, a higher central venous pressure target before tumor resection, or earlier transfusion threshold) and to avoid tachycardia or excessive inotropes. Similarly, pre-induction ultrasound assessment of the IVC could inform how volume-replete the patient was (many elderly patients are relatively dehydrated after overnight fasting and bowel preparation). Addressing a low circulating volume before major vascular ligation (like adrenal vein clamping) might have lessened the degree of hypotension encountered.

## Conclusions

This case report emphasizes the importance of comprehensive hemodynamic assessment in the perioperative management of large adrenal tumors. A rare adrenal cavernous hemangioma masqueraded as a malignant tumor and led to significant postoperative hemodynamic instability. An unnecessary exploratory laparotomy was performed due to concern for hemorrhage, exposing the patient to additional risk. The use of point-of-care ultrasound in the PACU (and even as part of preoperative evaluation) could have identified the true cause of hypotension - in this instance, likely hypovolemia and vasodilation - thereby avoiding a return to the operating room. POCUS is a powerful adjunct for anesthesiologists, allowing rapid, bedside differentiation of shock states and assessment of volume status and cardiac function. Its application in this case might have guided more tailored resuscitation and prevented the cascade of complications associated with re-operation. We recommend that anesthesiology teams caring for patients with large tumors or equivocal hemodynamics strongly consider employing POCUS to inform clinical decisions. By integrating ultrasound into perioperative care, providers can enhance patient safety, optimize management, and potentially avert avoidable procedures. This case highlights how modern perioperative ultrasound technology can improve outcomes and underscores the need for vigilance and advanced monitoring when assumptions (such as presumed bleeding) are not yet confirmed.
